# GABA Signaling: Therapeutic Targets for Neurodegenerative and Neurodevelopmental Disorders

**DOI:** 10.3390/brainsci13091240

**Published:** 2023-08-25

**Authors:** Enrico Cherubini, Yehezkel Ben-Ari

**Affiliations:** 1 European Brain Research Institute (EBRI), Rita Levi-Montalcini Foundation, Viale Regina Elena 293-295, 00161 Roma, Italy; 2 Neurochlore, Campus Scientifique de Luminy, 163 Route de Luminy, CEDEX 09, 13288 Marseille, France; ben-ari@neurochlore.fr

This Special Issue, “GABA Signaling: Therapeutic Targets for Neurodegenerative and Neurodevelopmental Disorders”, focuses on a fundamental property of the neurotransmitter γ-aminobutyric acid (GABA), namely its capacity to shift, in particular conditions, from the hyperpolarizing to the depolarizing direction.

GABA, the main inhibitory neurotransmitter in the adult brain, hyperpolarizes the membrane and inhibits neuronal firing by activating two different classes of receptors: GABA_A_ and GABA_B_. Unlike GABA_A_ receptors, which form integral ion channels, GABA_B_ receptors are coupled to ion channels via guanine nucleotide-binding proteins and second messengers. In physiological conditions, GABA exerts a powerful control over cell excitability and network oscillations thought to be associated with high cognitive functions.

Early in postnatal life, GABA, acting on GABA_A_ receptors, depolarizes and excites its targets through an outwardly directed flux of chloride. The low expression of the KCC2 extruder at birth leads to Cl− accumulation inside the neuron via NKCC1. The developmentally up-regulated expression of KCC2, which in rodents occurs toward the end of the first postnatal week, results in the extrusion of Cl^−^, causing a shift of GABA from the depolarizing to the hyperpolarizing direction. Therefore, the direction of GABA_A_-mediated neurotransmission (depolarizing or hyperpolarizing) depends on the intracellular levels of chloride [Cl−]i, which in turn are maintained by the activity of the cation-chloride importer and exporter: NKCC1 and KCC2, respectively [1]. This system is highly labile and is altered in a wide range of brain disorders, leading to a reduction in KCC2 with the consequent accumulation of chloride inside the cell, and a depolarizing and often excitatory action of GABA. These alterations have been observed not only in developmental disorders, but also in neurodegenerative ones, as well as in trauma, infarct, and lesions, suggesting that this is a common reaction of networks to insults.

This research topic comprises one research and four review articles, highlighting the role of GABA neurotransmission in several brain disorders and the beneficial effect of restoring proper GABAergic signaling and excitatory/inhibitory balance in the neuronal circuits involved.

The research article by Alfano et al. focuses on the function of GABA_A_ receptors in type II focal cortical dysplasia (FCD), one of the most frequent drug-resistant forms of epilepsy in pediatric patients [2]. Using *Xenopus oocytes* transplanted with human tissues from patients affected by FCD, the authors found that while the prototypical pro-inflammatory cytokine IL-1β reduced the amplitude of GABA responses via its signaling pathway in tissues obtained from adults, it potentiated those obtained from children. In agreement with previous data, this effect was associated with a more depolarizing response to GABA, caused by the upregulation of the chloride importer NKCC1 [3]. These results indicate that an inflammatory process and an altered chloride homeostasis are responsible for the enhanced excitability and focal epilepsy induced by cortical dysplasia in children. Whether the two processes are causally related remains to be established.

Emerging non-invasive proton magnetic resonance spectroscopy (^1^H-MRS) used to unveil the role of GABAergic signaling in addictions caused by alcohol, nicotine, cocaine, and cannabis has been reviewed by Shyu et al. [4]. Improvements in this technique have allowed the quantification of GABA concentration and the monitoring of its dynamic changes associated with addiction in order to better characterize the nature of this disorder and its treatment using drugs acting on GABAergic transmission. Changes in GABAergic signaling are effective in reducing withdrawal, cravings, and other addiction-related behaviors such as stress, emotion, memory, attention, and reward processing. However, most studies are still preliminary, based on a relatively small sample size, and do not reach clinical significance. Moreover, whether changes in GABA concentration following different addictive substances are region-dependent remains to be determined. Although still in its infancy, MRS appears to be a promising non-invasive method to quantify in vivo metabolite concentrations in addiction research.

In their review, Szymanski and Minichiello discuss how, early in postnatal development, brain-derived neurotrophic factor (BDNF) and the TrkB signaling pathway alter the expression of the cation-chloride importer NKCC1 [5]. As reported in a recent paper by Badurek et al., the specific deletion of *Ntrk2/Trkb* from immature mouse hippocampal dentate granule cells, during a particular time window, impairs their integration in the proper hippocampal circuitry and reduces the expression of NKCC1 in their targets, the CA3 principal cells [6]. The premature shift of GABA from the depolarizing to the hyperpolarizing direction leads, at the network level, to the disruption of early synchronized neuronal activity and to the impairment of the morphological maturation of CA3 pyramidal neurons, ultimately contributing to alter the hippocampal synaptic plasticity and cognitive processes in adulthood. A premature shift in GABA from the depolarizing to the hyperpolarizing direction, associated with reduced BDNF/TrkB signaling, has recently been found in a mouse model of autism spectrum disorders, carrying the human R451C mutation of the *Nlgn3* gene (NLG3^R451C^ KI) found in some families with autistic children. Similar results were obtained in mice lacking the *Nlgn3* gene (NLG3 KO mice), suggesting a loss of function [7].

The expression of GABA_B_ receptors in the developing brain has been reviewed by Bassetti [8]. By modulating the function of K^+^ and Ca^2+^ channels, these receptors, expressed at both post and pre-synaptic levels on glutamatergic and GABAergic terminals, influence several developmental processes including cell migration during cortex formation, cell maturation, and network development. They are also expressed in astrocytes, where they are instrumental in shaping neuronal activity and plasticity processes. Many interacting proteins may participate in the formation of GABA_B_ receptor complexes, leading to a variety of different functions which can be targeted by newly designed drugs. GABA_B_ receptors are developmentally regulated; they are already present at embryonic stages, and their distribution varies during development in a region-specific manner. Their dysfunction may affect brain growth and circuit formation. Thus, GABA_B_ receptors represent a suitable target for treating a variety of developmental brain disorders.

Finally, the review by Capsoni et al. highlights the role of GABA_A_-mediated neurotransmission in neurodegenerative disorders such as Alzheimer’s disease (AD) [9]. Alterations in nerve growth factor (NGF) signaling, discovered in 1952 by Rita Levi-Montalcini and primarily involved in the growth, maintenance, proliferation, and survival of peripheral and central neurons, play a key role in AD development. Previous data have reported changes in GABAergic signaling and alterations in the excitatory/inhibitory balance in hippocampal neurons, as well as in the role of the cation-chloride co-transporters in mice engineered to express recombinant neutralizing anti-nerve growth factor (NGF) antibodies, which develop a neurodegenerative pathology reminiscent of that observed in AD patients. In the hippocampus of these mice, GABA exerts on its targets a depolarizing and excitatory action due to the intracellular accumulation of chloride, consequent to a reduction in the cation-chloride exporter KCC2, an effect that can be reversed by bumetanide, a selective antagonist of the cation-chloride importer NKCC1 [10]. An impressive new therapeutic approach, consisting of the repurposing of bumetanide, the selective antagonist of the cation-chloride importer NKCC1, has recently been provided to treat late-onset AD [11]. Bumetanide ranked 1st out of 1300 compounds as the most effective flipping drug, and it was selected as the top candidate for further investigations because of its safety in long-term use and its positive effects on other brain diseases. Perhaps more impressively, analyzing millions of clinical files, the authors also show that adults (>65 years old) treated with bumetanide have a significantly reduced probability of developing Alzheimer’s disease. The therapeutic potential of bumetanide for AD treatment was validated both in vitro and in vivo and statistically with real-world data.

We wish to thank all authors who submitted their work to this Special Issue and the reviewers for dedicating their time to improving the quality of the published manuscripts.
